# A New Long-Term Reversible Contraception Method: Sexual and Metabolic Impact

**Published:** 2012-10-11

**Authors:** F Visconti, F Zullo, M.L Marra, G De Masellis, M Caiazza, F Cibarelli, B Buonomo, M. Guida

**Affiliations:** 1 Department of Gynecology and Obstetrics, University of Salerno, Salerno, Italy; 2 Family Planning Clinics of Battipaglia, Salerno, Italy; 3 Family Planning Clinics of Salerno, Salerno, Italy

## Abstract

**Background:**

Data relating to the influence of hormonal contraception on sexual life are conflicting and mostly they refer to oral contraceptives. In this study we evaluated the effect of a long-acting contraceptive on sexual function, metabolism and bleeding pattern variations.

**Methods:**

23 women with a permanent partner and an active sexual life completed a specific questionnaire at the start of the study and after cycles 3 and 6 of contraceptive use; a blood sample was performed or metabolic evaluation and a “bleeding calendar” was compiled by the patients.

**Conclusion:**

There is an increase of quality and frequency of sexual function after 6 month of contraception; there aren’t significant change in metabolic parameters and is detectable a modification of bleeding patterns.

## I- INTRODUCTION

Fifty years after their introduction, hormonal contraceptives still represent the most effective reversible method for family planning. In recent years, to reduce side-effects associated with the contraceptive administration, the dosage of ethinylestradiol (EE) has been gradually reduced from 50 mg to 20–15mg and new progestogens have been introduced with a reduced androgenic activity and low side-effects. The mostused hormonal contraceptives are administered by the oral route and need daily administration, 21–24 days per cycle [1].

However, women have recently shown an increased interest towards hormonal contraception that does not require daily intake.

Recently a new subcutaneus implant relasing etonogestrel (ENG) for long acting contraception has been developed [2], which presents a cylindrical rod structure, flexible and biodegradable, that contains 68 mg of ENG.

The new device is intended to totally replace the Implanon®, which is the most widely used implantation system in the world.

From a pharmacological point of view, the Nexplanon® is equivalent to Implanon® [3]. In fact, both contain 68 mg of ENG and, once placed at the sub-dermal level, provide contraceptive coverage to women of at least 3 years; however, significant different exist between the two devices both in the implant structure and in the procedures required for its insertion [4].

Women who use a contraceptive method wont to get have a higher frequency and a higher quality of sexual intercourse.

Data related to the influence of hormonal contraception on sexual life are conflicting and they refer to oral and intravaginal contraceptives: as no studies are present in the literature on Nexplanon influence on users sexual life, we decided to develop a study to analyze this important aspect.

## II - MATERIALS AND METHODS

All women who came to the Contraception’s Center of our Department, from 1 June 2011, were asked to participate in the study for the valuation of hormonal contraceptive influence on users’ sexual activity, metabolism and bleeding problems.

The participants had to be between 20 and 45 years of age, have a Body Mass Index between 19.5 and 26.6 kg/m^2^, have an active sexual life and a regular menstrual cycles; we included women who have had a voluntary interruption of pregnancy and women that frequently occurring at emergency contraception.

Exclusion criteria included confirmed pregnancy or suspicion thereof, evidence of acute, chronic or progressive hepatic disease, evidence of vascular disorders, evidence of malignancies, undiagnosed vaginal bleeding, use of drugs known to influence the pharmacokinetics of contraceptive steroids and all other clinically relevant contraindications of the use of hormonal contraception.

Before inserting the contraceptive, all the women provided a medical and gynecological history and they were carry out a gynecological examination, Papanicolaou test, vaginal buffer, evaluation of blood pressure, calculation of the BMI and complete haematochemical tests.

Twenty three of forty five women- who inserted the contraceptive- have reached the study period of six months.

Nexplanon has been inserted between the first and the fifth day from the beginning of the menstrual cycle. The effect of ENG contraceptive implant on the sexual activity of patients has been assessed by a specific test: the Interviewer Ratings of Sexual Function (IRSF) which evaluates some psychological and physical aspects of sexual interactions [5]. The IRSF is a semistructured interview involving ten items, and the women have to rate their score for each question on a visual analogue scale (VAS) of 100 mm.

The IRSF was carried out on the women at the baseline, after 3 and 6 months by the introduction of the BMI and the duration of partners’ current relationships showed a normal distribution, and differences between groups at the beginning of the study for these variables were evaluated by Student’s t-test for unpaired data.

The data for level of education, number of children and contraceptive.

The calculated differences in VAS score (ΔVAS) between baseline (VAS0), month 3 (VAS3) and month 6 (VAS6) were used to evaluate the effects of the contraceptive use on sexual function for each patient for each item investigated by the questionnaire. The calculated differences were:

ΔVAS1=VAS3-VAS0;ΔVAS2=VAS6-VAS0 and ΔVAS3=VAS6-VAS3.

The effect of ENG contraceptive implant on the metabolism was performed a venous blood sample on the women at the baseline (MET0), after three (MET3) and six (MET6) months by the introduction of the contraceptive.

For each metabolic parameter evaluated was calculated the difference (ΔMET) in three different moments:

ΔMET1=MET3-MET0;ΔMET2=MET6-MET0 and ΔMET3=MET6-MET3.

The effect of ENG contraceptive implant on bleeding pattern was performed through a “bleeding calendar”; to assess the participants’ menstrual pattern, they were instructed to record the presence of absence of bleeding. In addition, weekly frequency variation of sexual activity, BMI variation and injuries at the site of insertion of the contraceptive (secondary outcome) of all patients were evaluated.

The Shapiro–Wilk’s test was performed to evaluate the distribution of data for all parametric variables. Age, methods of contraception previously used showed a non-normal distribution and therefore the differences between the groups for these variables were calculated by Mann–Whitney test.

The differences between the three groups in mean ΔVAS and in baseline VAS score were statistically evaluated by Mann–Whitney test for independent variables.

BMI and weekly frequency of sexual activity were evaluated in two limes: at baseline and after six months, the statistical significance was evaluated by t-test for paired data.

Statistical significance was set at P, 0.05.

## III - RESULTS

The Study group consisted of 23 women; only one patient discontinued the contraceptive use for a negative effects on libido and galactorrhea (uncommon side effect).

There were no differences between our patients at baseline in terms of age, BMI, duration of relationship with partner, level of education, number of children, previous contraceptives used and weekly frequency of sexual activity.

At the baseline there were no significant differences between the patients in terms of parameters investigated by IRSF; the modification of these parameters in patients during the six months is reported in Table 2.

Improvement of sexual function in women after 3 months of contraceptive insertion demonstrated by the increase of some parameters expressing positive sexual function (frequency and intensity of orgasms, satisfaction) and by the decrease of one parameter expressing negative sexual function (anxiousness).

Improvement of sexual function in women after 6 months of contractive insertion demonstrated by the increase of sexual pleasure, personal initiative, frequency of orgasm and satisfaction, and by the decrease of discomfort and anxiousness.

At the baseline there were no significant differences between the patients in terms of metabolics parameters investigated; the modification of these parameters in patients during the six months is reported in Table 3.

By statistical analysis of metabolic parameters showed that after 3 months of contraceptive insertion there is an increase of glycemia and activity of prothrombin and a decrease of AST, ALT, total cholesterol and triglycerides; after 6 months of contraceptive insertion there is an increase of activity of prothrombin and is conformed the decrease of the parameters mentioned above (Tab. 2). All variations falling within the normal range.

Furthermore, in our patients there was an increase in weekly frequency of sexual activity and a reduction in body weight after 6 months of contraceptive insertion (BMI p-value: 0.013; weekly frequency of sexual activity p-value: 0.003).

The 60.90% of women reported unwanted skin reaction at the site of insertion of the contraception (swelling, itching and bruising) but the discomfort experienced was minor, with disappearance in 7 days.

At the end, by the evaluation of the “bleeding calendar” was found that about the 50% presents a reduction of blood flow and the remaining part has an increase of blood flow.

## IV - DISCUSSION

Fifty years after their introduction, hormonal contraceptives still represent the most effective reversible method for family planning. In recent years, to reduce side-effects associated with the contraceptive administration, the dosage of ethinylestradiol (EE) has been gradually reduced and new progestogens have been introduced with a reduced androgenic activity and low side-effects.

However, the development of these formulations of estrogen-progestin does not take into account of the effects on behavior, mood and sexuality of the woman who takes them.

But do not forget that compliance is as comprehensive concept of acceptability, not depends only on a neutral metabolic performed by the pill, but also of sexuality individual and the couple: the success of contraception depends on the changes that sexuality undergoes, in the course of hormonal contraception, in fact, the woman would expect an improvement in the their sex lives now private anxiety of an unwanted pregnancy, and in this scenario that the effectiveness measured through its effect on sexuality, he takes the role of central pillar of contraception.

Some studies showed that women who take oral hormonal contraceptives have a higher frequency of sexual intercourse compared to women who use other contraceptive methods, with an increased frequency and intensity of orgasms [6] while others have shown that other contraceptives are associated with negative effects on libido and reduced women’s sexual activity [7].

Our study showed that users of a long- acting contraceptive method, show an increase in both the coital frequency that an improvement of its quality.

These results can be explained in part by a significant increase of the complicity of couple: culture, education and social environment affect a woman’s sexuality, also, must be considered boung at the psychological effect the use of a high contraceptive efficacy as the Nexplanon, which influences sexual activity including through greater psychological availability.

## Figures and Tables

**Table 2:** Effects of contraceptive use on sexual function

Evaluation parameter of sexual function	Visual Analogic Score					
	ΔVAS 1	p-value	ΔVAS 2	p-value	ΔVAS 3	p-value

- Sexual pleasure	−7.8±25.7	0.159	−25.7±12.7	**0.002**	−7.1±14.9	0.253
- Pain	4.3±21.5	0.343	−5.7±19.9	0.476	2.8±4.9	0.172
- Personal initiative	−6.5±34.2	0.370	−31.4±10.7	**0.000**	−31.4±23.4	0.012
- Sexual Interest	1.7±29.2	0.778	−4.3±29.9	0.718	0±5.8	1.000
- Sexual Fantasies	4.3±26.9	0.447	−21.4±13.4	0.006	−7.1±9.5	0.094
- Orgasm	−13.0±22.8	**0.012**	−28.6±21.9	**0.014**	−2.8±7.5	0.356
- Intensity of orgasm	−15.2±24.8	**0.008**	−30.0±24.5	0.018	1.4±3.8	0.356
- Satisfaction	−11.7±20.6	**0.012**	−31.7±11.7	**0.001**	−8.4±24.0	0.434
- Complicity	−0.4±18.4	0.991	−8.3±24.0	0.434	2.8±5.00	0.173
- Discomfort	16.0±39.9	0.067	25.7±22.9	**0.025**	2.8±7.5	0.356
- Anxiousness	42.6±35.7	**0.000**	48.6±31.3	**0.006**	11.4±20.3	0.188

Data are expressed as mean ± SD

ΔVAS= difference in visual analogue score (for full definitions see Materials and methods).

**Table 3:** effects of contreceptive use on metabolics parameters

Metabolics parameters	Metabolic Score					
	ΔMET1	p-value	ΔMET2	p-value	ΔMET3	p-value
Glycemia	−16±8.8	**0.000**	−13±16.2	0.077	6.1±15.8	0.342
AST	6.1±15.8	**0.000**	8.3±3.5	**0.000**	1±1.41	0.110
ALT	7.6±6.5	**0.000**	9±1.7	**0.000**	0.8±2.3	0.355
LDH	13.5 ±74.9	0.397	5.6 ±63.3	0.823	1±53.9	0.962
Total cholesterol	17.9±17	**0.000**	20.4±6.1	**0.000**	5.8±7.9	0.097
Triglycerides	26.5 ±27.5	**0.000**	45.1±15.8	**0.000**	14±13.7	0.035
Activity of prothrombin	−8.1±10.5	**0.001**	−13.2±12.3	**0.028**	−5.7±8.7	0.134
Prothrombin time	0.1 ±0.5	0.419	0.1±0.4	0.664	−0.2±0.5	0.292
I.N.R	0.0±0.1	0.129	0.0±0.1	0.779	0.0±0.1	0.960
aPTT	2.1±4.5	**0.032**	−0.3±4.7	0.864	−0.9±4.1	0.598
aPTTratio	0.1±0.2	0.080	0.1±0.1	0.101	0.0±0.2	0.782
Derived fibrinogen	2.2±47.7	0.832	−31±39.6	0.083	−24=53.6	0.281

Data are expressed as mean ± SD
